# Investigating the hexavalent chromium removal from aqueous solution applying bee carcasses and corpses modified with Polyaniline

**DOI:** 10.1038/s41598-021-97518-7

**Published:** 2021-09-27

**Authors:** Seyed Ali Hosseini, Majid Riahi Samani, Davood Toghraie

**Affiliations:** 1 Department of Civil Engineering, Khomeinishahr Branch, Islamic Azad University, Khomeinishahr, Iran; 2 Department of Mechanical Engineering, Khomeinishahr Branch, Islamic Azad University, Khomeinishahr, Iran

**Keywords:** Environmental sciences, Natural hazards

## Abstract

There are currently heavy metals in most industrial effluents which are among the most significant environmental pollutants. Hexavalent chromium is one of the most significant heavy metals. In this research for the first time, eliminating the hexavalent chromium from the aqueous medium/aquedia applying bee carcasses and corpses modified with polyethylene was examined. Adsorption experiments were conducted discontinuously on laboratory solutions, including hexavalent chromium. The optimal adsorption conditions such as different pH factors, contact time, initial chromium concentration, and adsorbent value on the adsorption rate were examined at different levels, and adsorption isotherms were plotted. Some adsorbent properties were examined using Field Emission Scanning Electron Microscopy, XRD analysis, Fourier Transform Infrared Spectroscopy, and BET test to study the properties of the synthesized adsorbent. This study indicated that the highest percentage of removal related to polyethylene composite and bee carcasses in the presence of polyethylene glycol was 50.56% among the bee carcasses composites. The parameters effective on the adsorption process for polyethylene composite and bee carcasses and losses in the presence of polyethylene glycol suggested that the adsorption percentage increased for this composite by decreasing the pH, increasing the contact time, and increasing the adsorbent. The highest percentage of adsorption was obtained when the pH was 2, the contact time was 120 min and the adsorbent value was 8 g/L and the initial concentration of chromium was 100 ppm. The most optimal removal percentage was achieved at the pH = 2, the contact time was 30 min, and the adsorbent value was 2 g/L, and the initial chromium concentration was 100 ppm. The results of drawing adsorption isotherms also indicated that higher R^2^ had a better fit than Langmuir for polyethylene composite and bee carcasses in the polyethylene glycol Freundlich equation.

## Introduction

Progressing the science and technology and the mechanization of human life and providing welfare and comfort for him/her have caused to enter dangerously and polluting substances into the environment^1,2^. Surface and groundwater are the main sources of clean water. Notwithstanding, many water resources have become polluted due to the rapid population growth and expanding industrial development in the world^3^. This can be due to the constant discharge of organic and mineral wastes from human activities in natural water resources^4^. We can refer to industrial effluents, including heavy metals, as one of the main and most significant environmental pollutants. Heavy metals are elements with a density higher than 3 g/cm^3^. Metal ions concentrated in the living body provide health and economic problems due to the biological toxicity of these contaminated ions^5–9^. Human resources to produce chromium include paints^10^, mining^11^, plating^12^, automobile manufacturing^13^, metal processing^14^, leather tannery^15^, and textiles manufacturing^16^. Chromium (VI) is more deadly than other metal ions and causes serious health problems at very high concentrations of 1000 mg/L while entering the body^17^. Consequently, it is required and essential to apply appropriate methods and develop new methods instead of the old chemical and physical methods to separate and remove them and purify water polluted with these heavy metals in today's society^18–21^. Repulsive forces between negatively charged materials and chromium (VI) ions or negative loads transfer chromium (VI) in the aquatic system and absorb chromium ions in the environment in soil and water environments. The concentration of chromium (VI) in groundwater and chromium-polluted wastewater worldwide is 30–200 mg/L^22^. Considering the higher toxicity of chromium (VI), the US-EPA^23^ institute has determined a safe chromium concentration of 0.1 mg/L in drinking water sources and 0.05 mg/L for home usages^24^. It can be absorbed through the respiratory system, skin, and gastrointestinal tract and has been recognized as one of the most significant human carcinogens, including lung cancer^25,26^. Chromium (VI) provides some side effects for humans such as asthma, irritation, and inflammation in the nose, contact dermatitis, deep wounds, piercing of the nasal septum, irritation of the lungs, skin allergies, and damage to the kidneys and liver^27–30^. At present, several methods are applied to remove and separate chromium from aqueous media^31–34^. Each one owns its advantages and disadvantages. For instance, it is not easy to recover the chromium in the chemical precipitation method because of producing a high amount of effluent^35^. The reverse osmosis method consumes a lot of energy and electricity and produces effluent^36^. Adsorption as a simple, environmentally friendly, and cost-effective method can eliminate different water pollutants than other techniques^37–42^. This adsorption process is a surface phenomenon^43–45^. Researchers and scientists currently seek to explore new attractions to separate and remove chromium and other heavy metals. It is possible to use wastes and other materials to achieve this objective in this regard^46–48^. The conductive polymers of polypyrrole and polyaniline and their composites are among the materials that have lately been recognized as adsorbents^49^. In such polymers, sensitivity to heat and air, conductivity, ease to moldability, solubility, and processability are highly different depending on the type of monomer and its synthesis method^50–52^. Polyaniline, a distinguished binding polymer, has been highly considered due to its ease of preparation, low price, and environmental stability^53^. Also, synthetic Polyaniline Composites (PANI) has multifunctional groups (active sites) and show high adsorption capacity for organic and inorganic pollutants^54–56^. Furthermore, this polymer was highly considered due to its electrical conductivity in the proper range^57^, many applications have been considered in various fields, and low cost, high environmental stability^58^. Much research has been conducted in this field around the world^28^. Other studies were examined the removal of the heavy metals using Paratoluene sulfonic/polyaniline nanocomposite^59^, polyaniline/sawdust/polyethylene glycol^60^, polyaniline/walnut shell^61^, polyaniline/zeolite nanocomposite^62^, Bacterial cellulose/polyaniline nanocomposite aerogels^63^. The results of this research have explained that various types of polyaniline and its composites can be synthesized by changing the synthesis conditions with absolutely different advantages and properties. This study, with a new idea and for the first time, aims to synthesize bee carcass composite and polyethylene modified bee wastes and apply them to separate chromium from aqueous media.

### Materials and methods

All applied materials did not require additional purification because of their high purity, except for monomer aniline, distilled and stored in the refrigerator before using until thoroughly discolored (because it is volatile and quickly destroyed and oxidized by air). All experiment stages used distilled water and were performed at 25 °C (PEG = 35,000 MW). All materials, including polyethylene glycol, aniline, potassium iodate, potassium dichromate, activated carbon, sulfuric acid, were employed, which were made by the German company Merck.

### Devices

Experiments were carried out using a pH meter PMT-Model 2002, XRD (PANALYTICAL X'PERT), a shaker machine, Field Emission Scanning Electron Microscope Machine (QUANTA FEG 450), Fourier transform spectrophotometer or Infrared spectrometer (FTIR) model TENSOR 27, UV–Vis Spectrometer device model GES30, BET device model Belsorp II made by BEL company.

### Synthesis methods

#### Preparing bee carcass and waste and polyaniline in the presence of polyethylene glycol

0.9 g of an oxidizing substance, including potassium iodate as an oxidant and 0.7 g of Polyethylene Glycol (PEG), was mixed in 150 mL of 1 M sulfuric acid solution obtained by combining 53.3 mL of pure sulfuric acid with 947 mL of distilled water in each experiment using a shaker. In another container, 1 g of bee carcasses and wastes were mixed with 1.5 mL of distilled aniline. It was transferred to the solution container and mixed on a shaker at room temperature for 5 h after soaking the contents of this container. The solutions were then removed from the shaker and passed through filter paper. The product was then washed several times with distilled water and dried in an oven at 50 °C for 48 h.

### The method to separate the Chromium using composites synthesized with bee carcasses and wastes

We provided a standard solution of 50 ppm chromium after providing the synthesized composites and adsorbents. A complete discontinuous mixing system was adopted to adsorb the heavy metal. Accordingly, 0.1 g of adsorbent (composites synthesized with bee carcasses and wastes) was added to 50 mL of 50 ppm chromium solution, and a shaker was used to mix it for 30 min with a shaker (magnetic stirrer) at a specified speed of 120 rpm. Then, we passed the solution through filter paper and the chromium concentration in aqueous media was determined by using the UV–Vis spectrophotometer^28^. The parameters regarded in this study include the amount of used adsorbent, contact time for perfect mixing, pH, and initial concentration of chromium.

### Freundlich and Langmuir adsorption isotherms

Freundlich and Langmuir's isotherms are proposed as follows:

1 $$X = x/m = \, KC_{e}^{1/n}$$

2 $$1/X \, = \, 1/X_{m} + 1/ \, \left( {b \, X_{m} C_{e} } \right)$$

and:

3 $$\log \, X/m \, = \, \log \, K \, + \, 1/n \, \log \, C_{e}$$

C_e_ is the concentration of metal in the liquid phase after mixing time (mg/L), n and k are Freundlich constants, X is the amount of adsorbed material per unit mass of adsorbent (mg/g), which indicated the intensity and capacity of adsorption, respectively. b is the Langmuir constant, which indicates the absorption energy, and X_m_ is the maximum amount of adsorbed material per unit mass of adsorbent (mg/g). In this study, different amounts of adsorbent were added to a solution of 50 mg/L chromium (VI) at ambient temperature.

### Investigating the chromium recovered by Polyaniline

0.1 g of synthesized polymer (composites synthesized with bee carcasses and wastes) was added to 150 mL of chromium-containing solution at a concentration of 50 ppm, and a shaker (magnetic stirrer) was used to mix it for 30 min at 120 rpm. After mixing, the solution was passed through a sieve, and 0.1 M sulfuric acid of the total adsorbent left on the filter paper was added to 50 mL and mixed on a magnetic stirrer for 30 min. Presently, after mixing, we filtered the solution through a filter paper and measured the concentration of chromium ion in the solution. The total adsorbent left on the filter was then added to 150 mL of 50 ppm chromium in the next step and mixed for 30 min in a shaker (magnetic stirrer). After final mixing, we measure chromium ion as follows.

4 $$R = 100 \, C_{t} /\left( {C_{0} - C_{e} } \right)$$

C_0_ is the initial concentration of chromium, C_e_ is the final concentration of chromium after separation, and C_t_ is the chromium concentration in sulfuric acid solution 0.1 M in this equation.

## Results and discussion

### Investigating the chromium separation using bee carcasses and wastes and their composite with polyaniline

**Figure 1:**
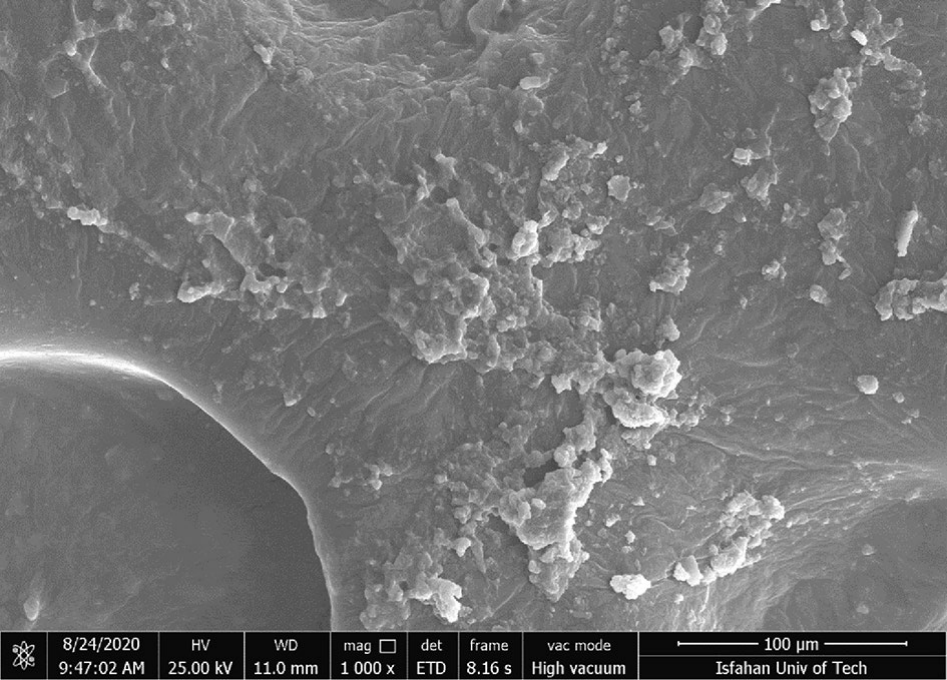
FESEM image of bee carcasses with 1000 X magnification.

**Figure 2:**
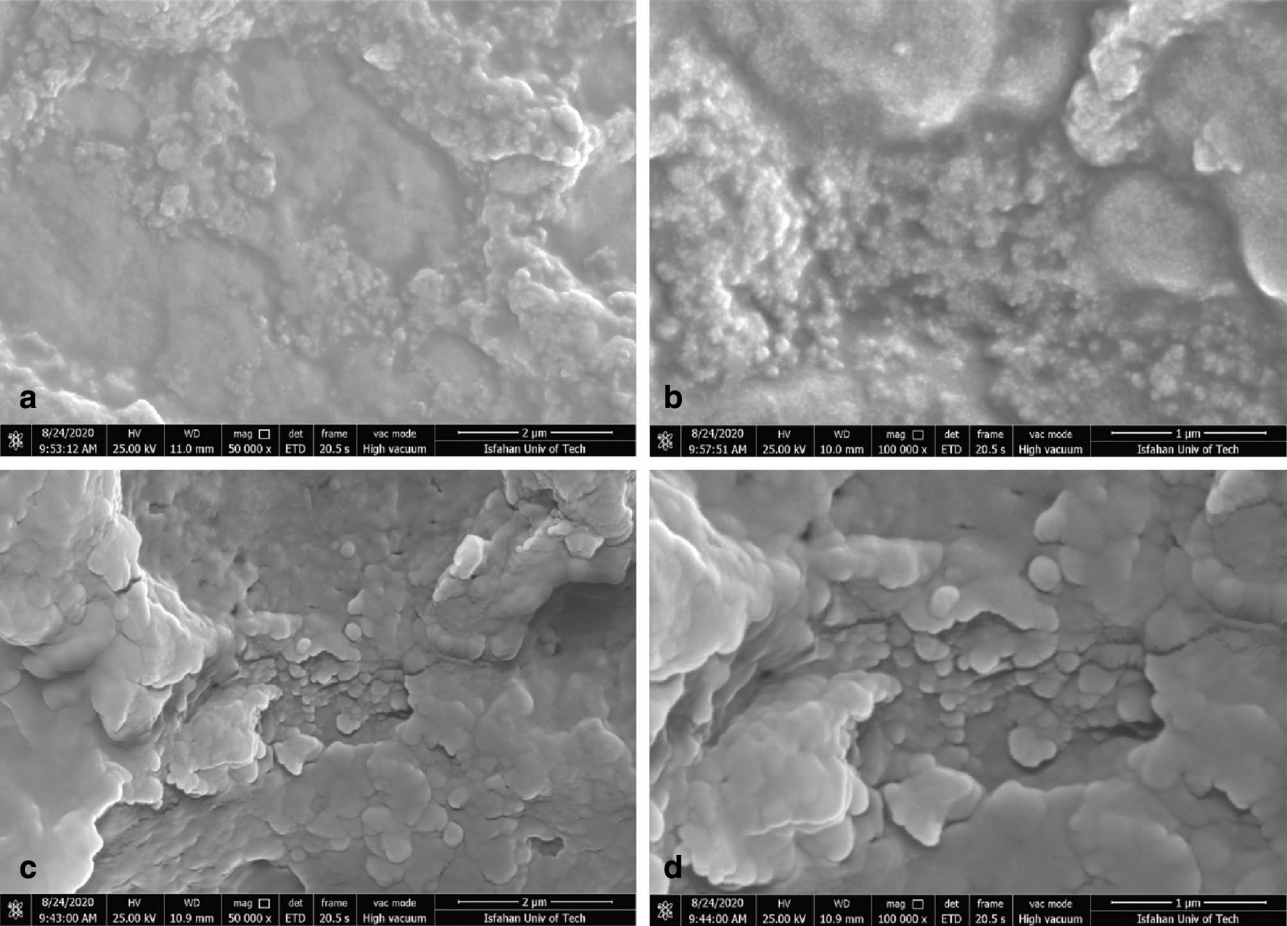
FESEM image of: (**a**) bee carcasses/polyaniline composite with 50000X magnification, (**b**) bee carcasses/polyaniline composite with 100,000 X magnification, (**c**) bee carcasses/polyaniline/polyethylene glycol composite with 50,000 X magnification, (**d**) FESEM images of bee carcasses/polyaniline/polyethylene glycol composite with 100,000 X magnification.

Table 1 shows the results of chromium separation using bee carcasses and wastes and their composites with polyaniline. As Table 1 explains, polyaniline coating has been empowered to affect the removal efficiency of all materials. The highest percentage of removal was related to bee-carcass composites with polyaniline in the presence of polyethylene glycol (4.6 g/L) with a removal percentage of about twice the activated carbon and 50.56% of removal among bee carcasses and wastes provided by polyaniline. Polyaniline coating on bee carcasses and wastes could increase the percentage of chromium removal. Earlier research and studies have explained that polyaniline owns the regenerative property of hexavalent chromium, and the presence of nitrogen (-NH) sites in polyaniline has absorbed chromium^64^. Figure 1 shows the results of field emission scanning electron microscopy for bee carcasses and wastes, and Fig. 2 (parts **a** and **b**) explains the results of bee carcasses and wastes with polyaniline. As this figure (parts a and b) show, Aniline is well coated on the surface of bee carcasses and wastes in polyaniline composite and bee carcasses and wastes, and polyaniline particles have been placed in the form of thin, irregular scales on the surface of bee carcasses and wastes.

**Table 1:** Results of chromium separation using bee carcasses and wastes and their composites with polyaniline (contact time 30 min, pH = 6, adsorbent amount 2 g/L).

No	Composite name	Initial Cr conc. (ppm)	Final Cr conc. (ppm)	Cr (VI) removal percentage (%)
1	Bee carcasses and wastes	50	38.24	23.52
2	Bee carcasses and wastes/polyaniline composite	50	37.02	25.96
3	Bee carcasses and wastes/polyaniline/polyethylene glycol composite	50	24.72	50.56
4	Activated carbon powder	50	37.12	25.76

According to Fig. 2**c** and **d**, the polyaniline particles are more uniformly and regularly and spherically covered on the surface of bee carcasses in the composite of polyaniline and bee carcasses and wastes in the presence of polyethylene glycol. Polyethylene glycol is a stabilizing material, and the adsorption of aniline particles has increased and has affected the shape and arrangement of the particles during the polymerization process. The achieved composite is more regular with a higher surface area, and a higher adsorption percentage has occurred. Table 2 shows the results achieved by analyzing the pore size and BET specific surface area for bee composite and wastes and polyaniline, bee composite and wastes, and polyaniline and polyethylene glycol. The results explain that polyaniline and polyethylene glycol have been increased the lateral surface of the composite in the carcass composite and bee wastes. Consequently, the removal efficiency has been increased.

**Table 2:** Results of BET analysis for bee carcasses and wastes and polyaniline composites.

Composite type	Special surface area (m^2^/g of adsorbent)
Bee carcasses and wastes/polyaniline composite	11.91
Bee carcasses and wastes/polyaniline/polyethylene glycol composite	16.72

**Figure 3:**
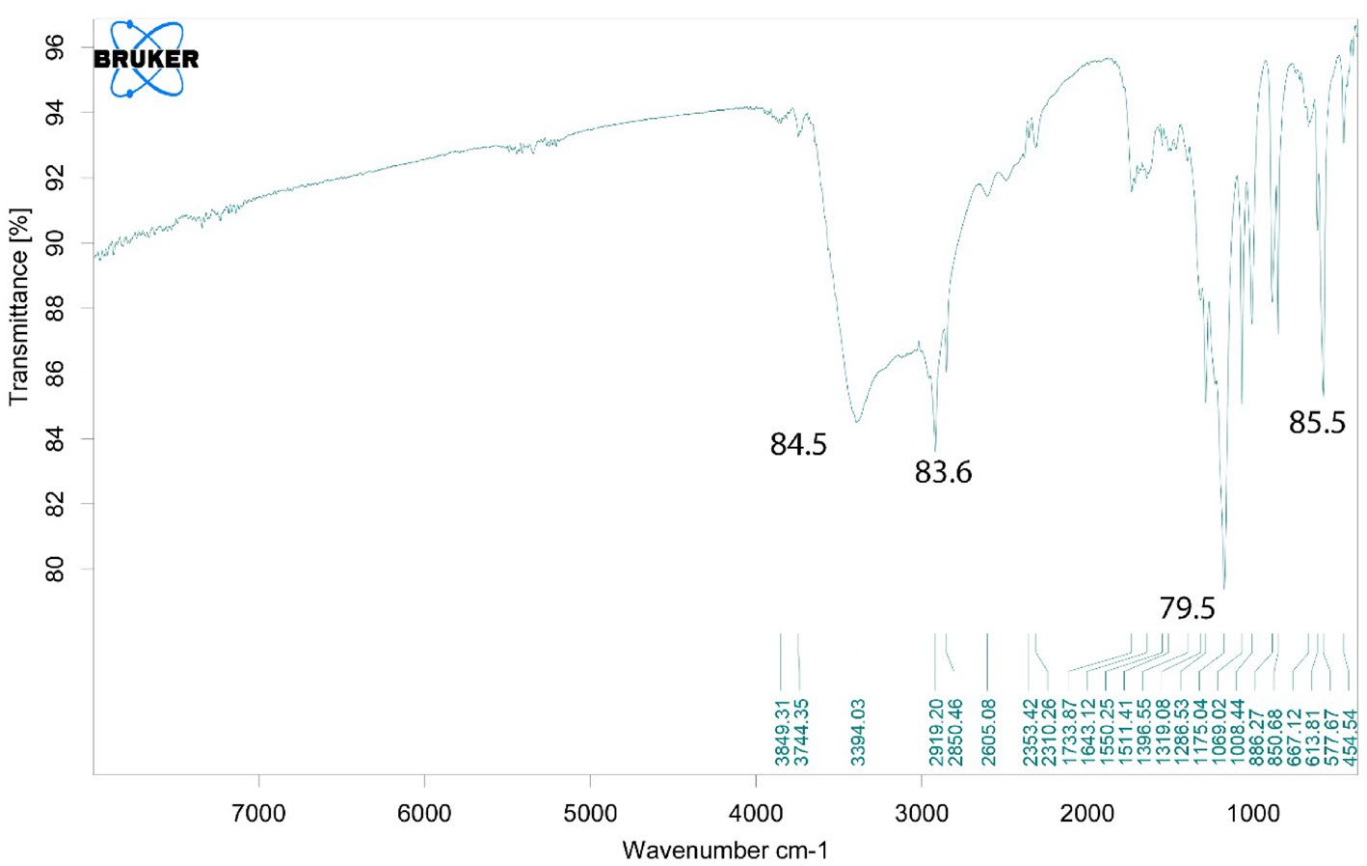
FTIR spectrum, related to bee carcasses.

**Figure 4:**
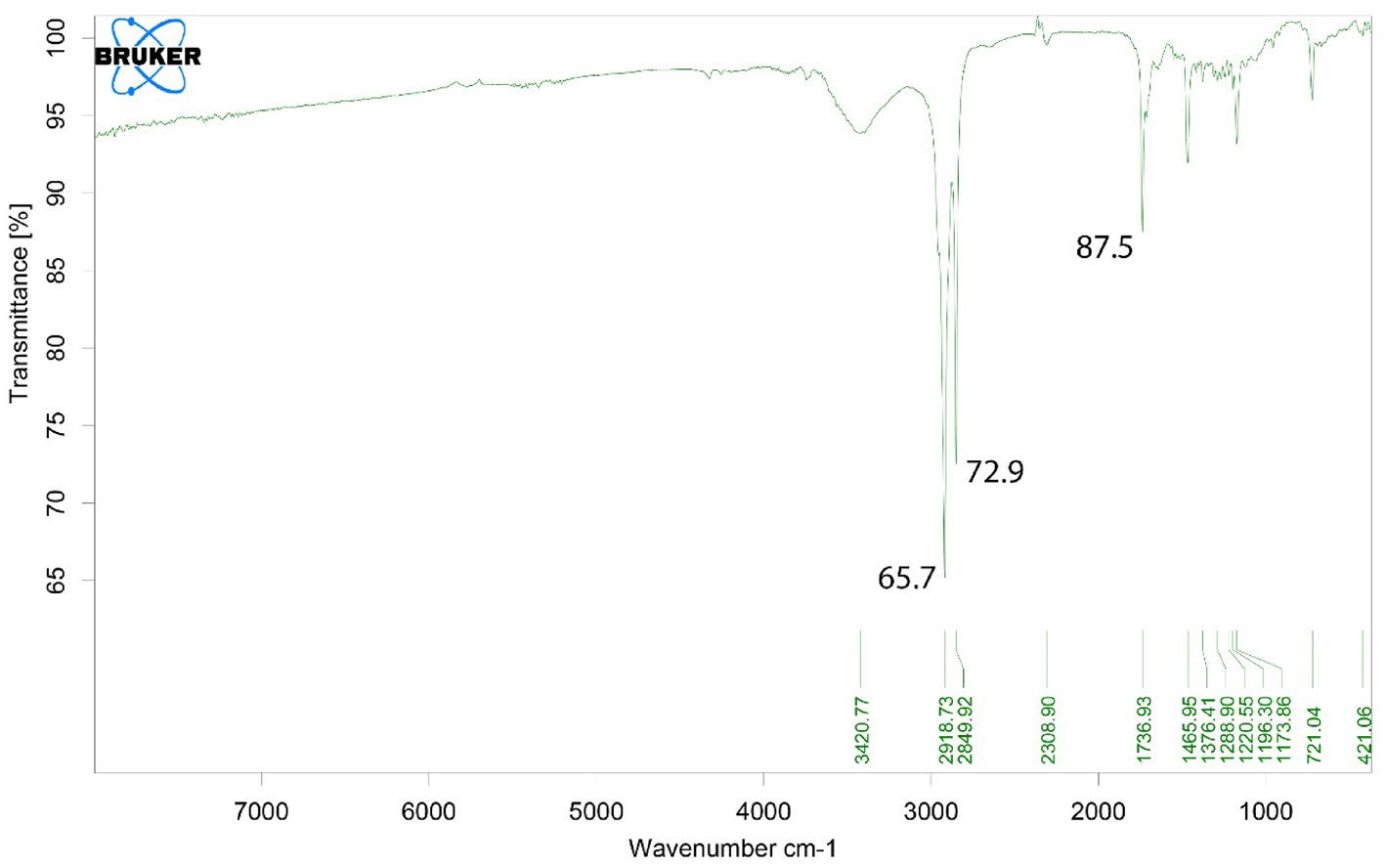
FTIR spectrum, related to bee carcasses/polyaniline/polyethylene glycol composite.

Figure 3 shows the FTIR spectrum of bee carcasses and wastes, and Fig. 4 shows the bee carcasses and composites and their polyaniline and polyethylene glycols. The deformation and location of the bands of the FTIR spectrum, with the different molecular environments, can be a helpful guide to the molecular structure of a compound^65^. As Figs. 3 and 4 show, the peaks recognized in 3400,2925,2110,1630,1193,1116,634 cm^−1^ belongs to functional groups such as tensile aromatic CH−, aromatic C=C−, alkyne C=C−, alkyl tensile CH−, or amine NH− and alcohol OH−.

**Figure 5:**
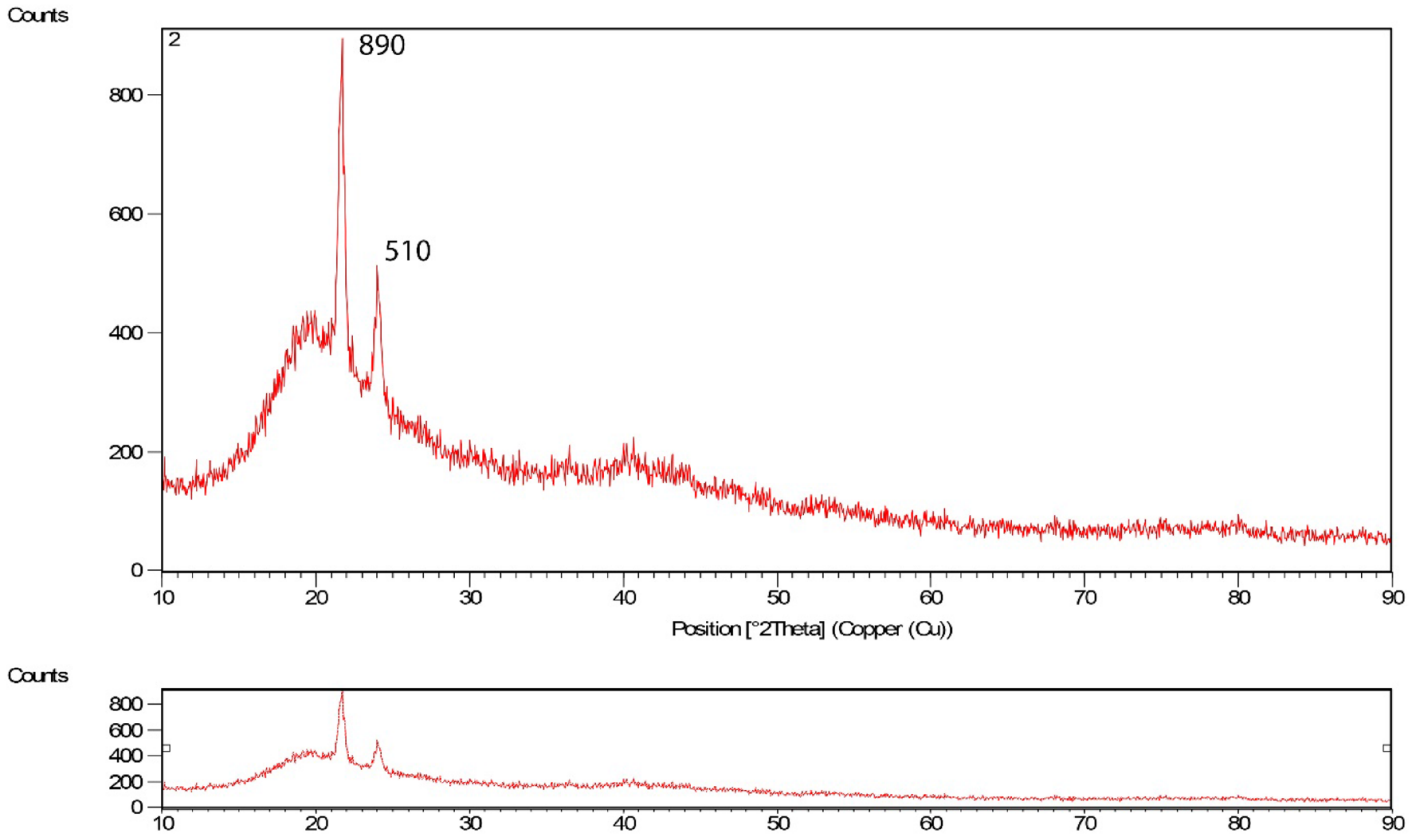
XRD pattern related to bee carcasses.

**Figure 6:**
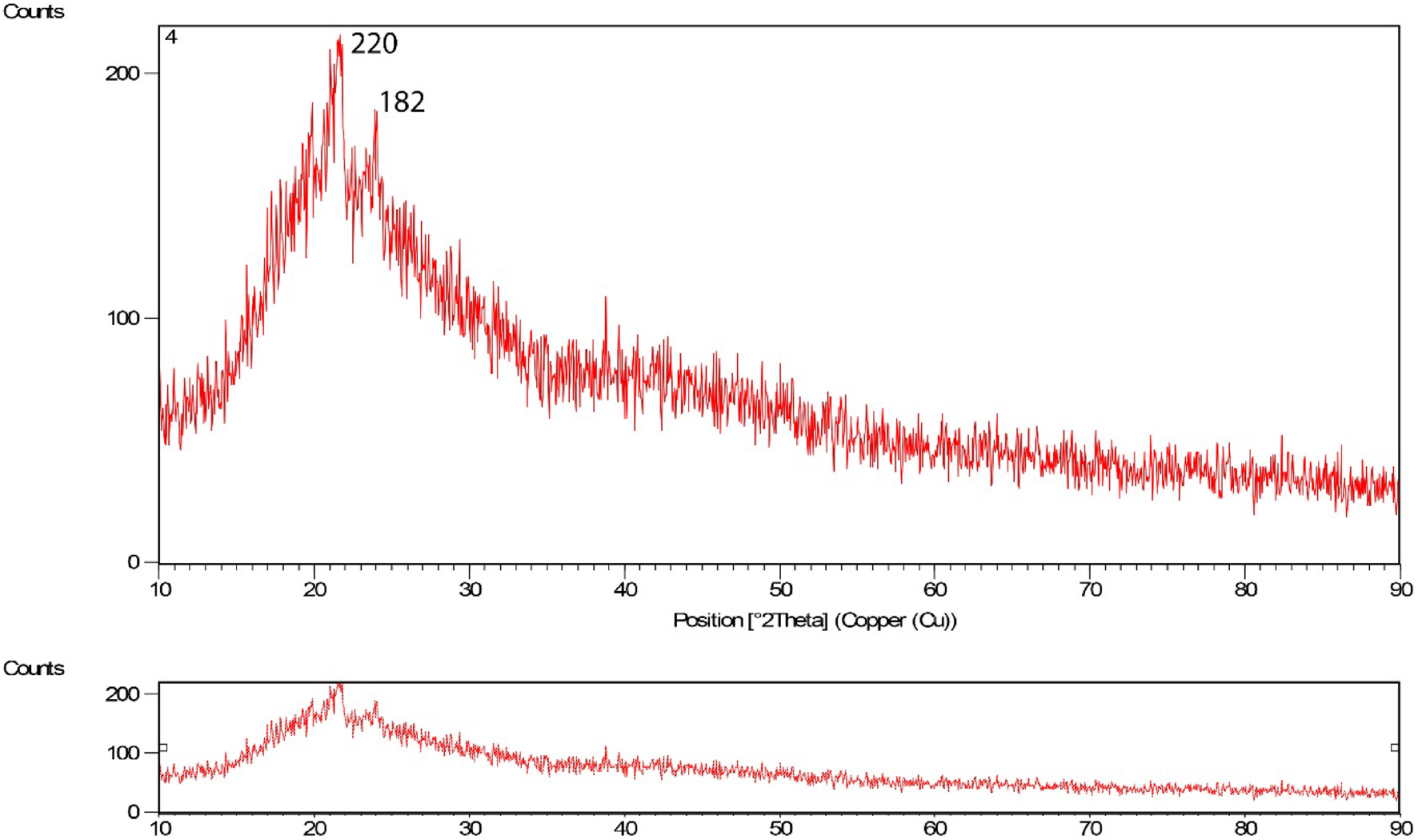
XRD pattern related to bee carcasses/polyaniline/polyethylene glycol composite.

Figure 5 shows the XRD results for bee carcasses and wastes. Figure 6 shows the polyaniline composite and bee carcasses and wastes in polyethylene glycol to determine the tested mineral properties of the adsorbents. As the analysis results accomplished on the synthesized composite explained, benzene compounds were observed.

### Examining the adsorption test conditions for bee carcass composite and waste/polyaniline/polyethylene glycol

#### Investigating the influence of pH of chromium solution on the efficiency of separation by bee carcass and wastes/polyaniline/polyethylene glycol

**Figure 7:**
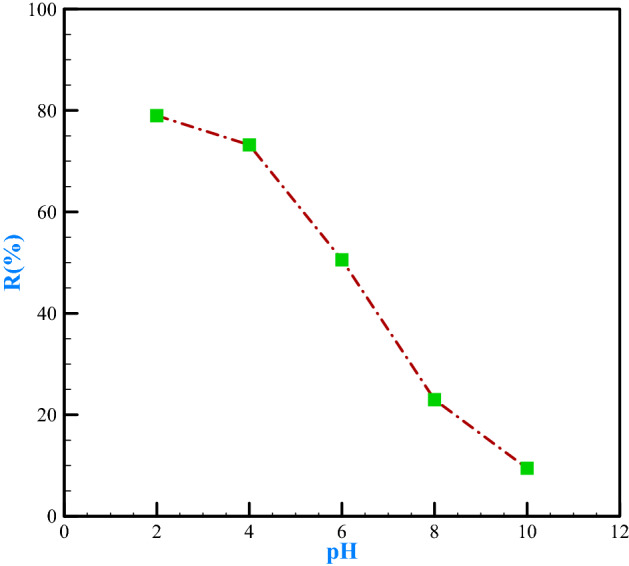
Removal efficiency versus pH.

pH is a factor that can enhance the adsorption efficiency and is one of the most significant and main parameters that determine the adsorption operation^59,66^. The pH influences biomass active site load and heavy metals behavior in solution^67^. Solution pH commonly impacts adsorbent ionization and adsorbent surface properties^68^. Nature of physicochemical interactions between adsorption sites and heavy metal ions to the pH of solution utilized in adsorption approaches, to a great extent^69,70^. in this research, five pHs (2, 4, 6, 8, and 10) were surveyed for chromium isolation and the results were indicated in Fig. 7. Alkaline pHs were avoided to prevent chromium sequestration.

The results reveal that the highest adsorption of chromium (VI) occurs at pH = 2 at 78.98%, and the lowest percentage of chromium (VI) adsorption happens at pH = 10 at 9.46%. The study results explained that the pH of the solution highly affects the amount of adsorption so that in most cases, increasing the pH decreases the amount of chromium adsorption. Changes in pH influence chromium adsorption because it determines the type of ionic species of chromium and the charge of the adsorbent surface. The predominant form at pH is 2 for Chromium + 6, and less than it, is as HCrO_4_^−^ and Cr_2_O_7_^2−^, its most value is HCrO_4_^−^, which is easily absorbed due to its low free energy. On the other hand, at high pH, the charge on the adsorbent surface is negative. Hence, the inclination to adsorb its anions decreases through the electrostatic process. According to increasing the adsorption at low pH and adding the potassium dichromate salt, chromium ions will be available in the form of Cr_2_O_7_^2−^ in the environment. Consequently, we can explain the decrease in adsorption by increasing pH because the –OH groups on the adsorbent based on Le Chatelier's principle tend to be transferred to the medium at low pH due to the low concentration of –OH ions in solution. Consequently, positive groups are formed on the adsorbent, which leads to adsorption, and –OH groups will no longer tend to be released on the adsorbent. The concentration pH will be increased in the solution by increasing the value of pH. Accordingly, fewer positive environments are created, and consequently, the absorption process occurs to a lower extent.

#### Examining the contact time

**Figure 8:**
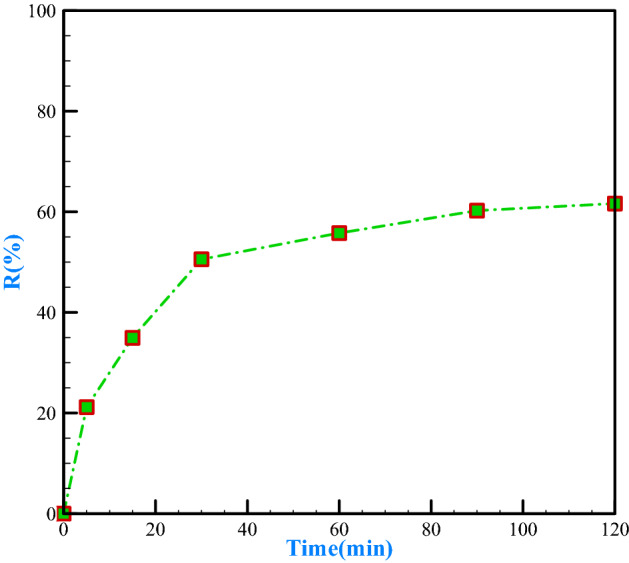
The effect of contact time on chromium removal.

The adsorption rate is frequently very high in the first stage of adsorption processes^71^. Figure 8 shows the effect of contact time of polyethylene composite sorbent and bee wastes in the presence of polyethylene glycol with solutions including chromium (VI) pH = 6. The metal concentration was 50 mg/L. The adsorbent was 2 g/L. Studies and researches conducted in this field explain that the amount and percentage of removal of the ascending path have been increased by increasing the contact time because many active and empty places in the adsorbent surface will be occupied over time^59,72^. And since, even after 120 min, the removal efficiency is still high, and there is no desorption process of the metal ions from the adsorbent at this time; therefore, the lowest amount of chromium removal was 21.16% in 5 min, and the highest amount of chromium removal was 61.64% in 120 min. Presently, the results achieved in the diagram show that the optimal contact time is 30 min, which is 50.56% for the removal rate, and from this time on, there is no significant removal rate.

#### Examining the amount of adsorbent

**Figure 9:**
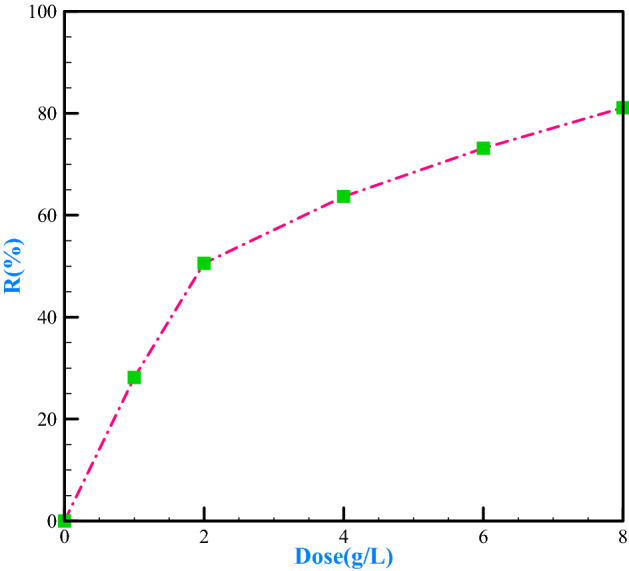
The effect of adsorbent amount dose on chromium removal.

The amount of adsorbent applied in removing metal ions is highly significant^73–77^. At this stage, we added different amounts of adsorbent (1, 2,4,6,8 g/L) to the chromium solution at pH = 6 and optimal contact time (30 min). As the results of Fig. 9 show, the removal efficiency was increased by increasing the amount of adsorbent. Polyaniline composite with bee carcass and waste in the presence of polyethylene-glycol could purify up to 81.1% of the solution, including 50 mg/L chromium in an adsorbent amount of 8 g/L. It is because of an increase in available places for the adsorption of metals, which has been performed by increasing the amount of adsorbent^46^. The results achieved in the diagram show that the optimal amount of adsorbent is 2 g per liter, which is a 50.56% removal rate, and the removal rate is then increased with a lower slope.

#### Examining the effect of initial chromium concentration

**Figure 10:**
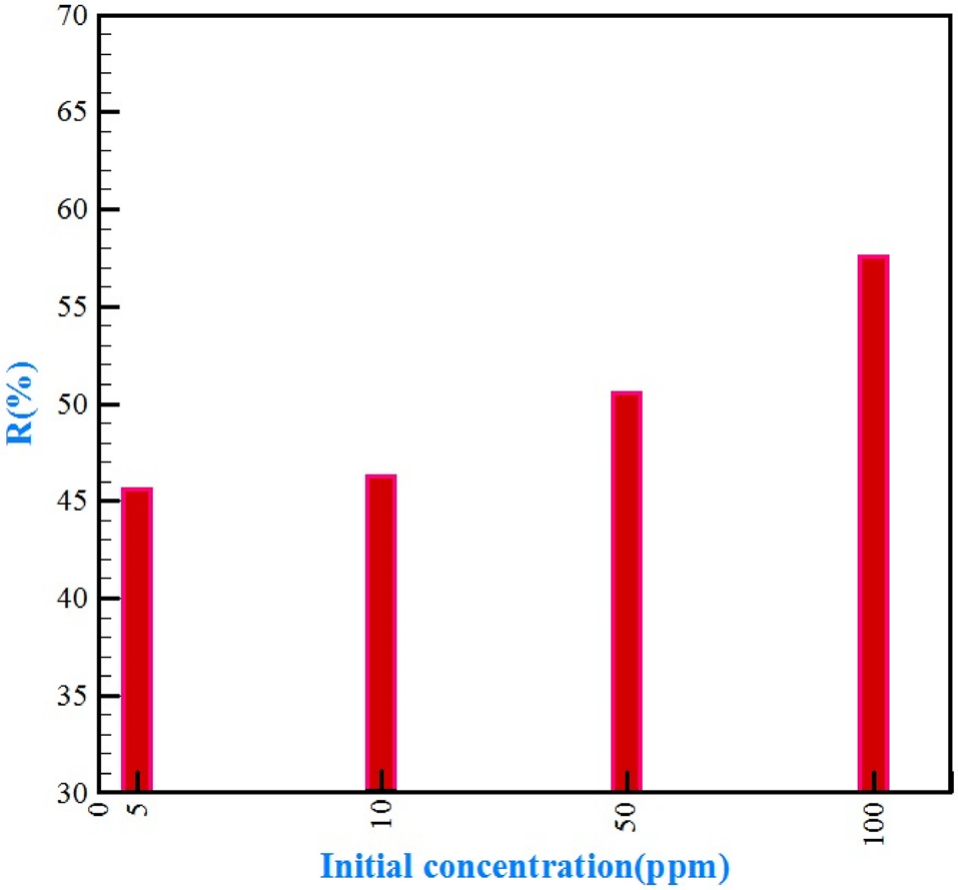
The effect of initial chromium concentration on separation efficiency.

A driving force is created in an aqueous solution with the initial concentration of sorbate, which helps to transfer Cr (VI) ions to the adsorbent surface^78^. At this stage, we examined the effect of initial concentrations of chromium (at four levels of 5, 10, 50, and 100 ppm) on the separation process by the selected composite. As the results of Fig. 10 show, polyethylene composite and bee carcasses and wastes in the presence of polyethylene glycol could remove the chromium optimally in the range of various concentrations of water and wastewater. And increasing the initial concentration of chromium from 5 to 100 ppm, the percentage of chromium removal from 45.63 to 57.59%, explains that the amount of adsorption was increased by increasing the initial concentration of chromium in the effluent. As the concentration of the solution increases, the density of ions in the solution increases. As a result, as the ions approach the surface of the particles, the adsorption percentage increases, consequently, the adsorption percentage is increased as the ions approach the surface of the particles, and consequently, the possibility of adsorption saturation of the metal ions increases^23, 79^.

### Langmuir and Freundlich adsorption isotherms for bee carcass composites and wastes/polyaniline/polyethylene glycol

**Figure 11:**
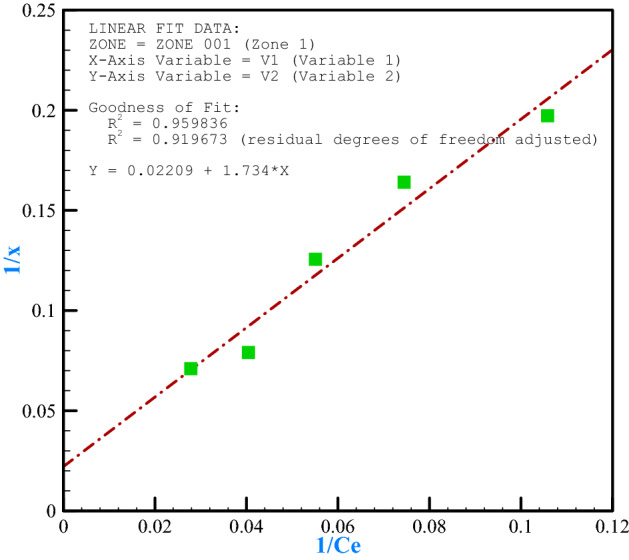
Langmuir isotherms for bee carcasses/polyaniline/polyethylene glycol composite.

**Figure 12:**
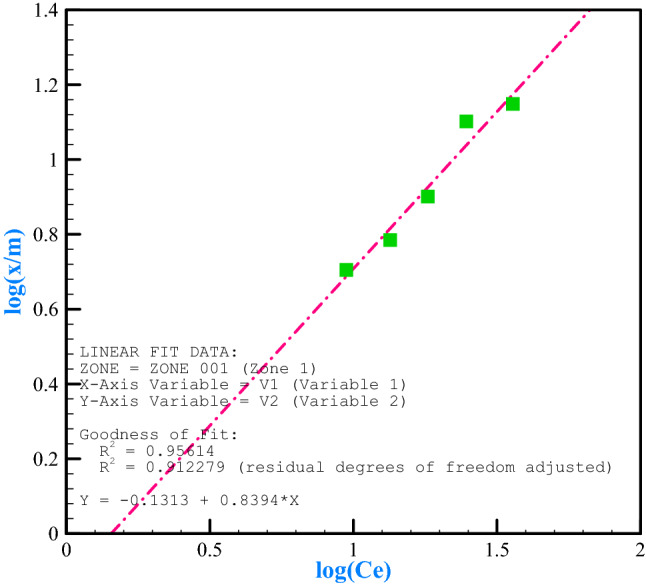
Freundlich isotherms for bee carcasses/polyaniline/polyethylene glycol composite.

The Langmuir and Freundlich adsorption models are usually used for absorption data^80^. We tested absorption data by Langmuir^81^ and Freundlich^82^ equations. At this stage, the adsorption isotherms were examined according to the values obtained before. Accordingly, Figs. 11 and 12 show the fit of the results with Langmuir and Freundlich adsorption models for chromium (VI). Table 3 also shows the parameters and constant coefficients of these models. Chromium metal fitted with both models due to the proximity of two R^2^s to each other, but it had higher compatibility in the Langmuir model due to higher R^2^ (R^2^ = 0.9598). The coefficient n of the Freundlich model, which is an indicator of adsorption intensity, was calculated at 1.19 for the adsorption of chromium by the adsorbent. The slope is increased by increasing the adsorbent concentration in this isothermal model but is eventually decreased to zero by filling the empty adsorption sites. The coefficient K is a measure of the absorption power in the Freundlich model, which is equal to 0.739 L per gram. In the Langmuir model, the R_L_ constant defines an important characteristic of this isotherm, named the equilibrium parameter. We calculated R_L_ values achieved for chromium adsorption by 0.611, which is between 0 and 1, and indicates the optimal adsorption to interpret metal adsorption by adsorbent^83^. The maximum adsorption capacity for chromium metal (X_m_) was 45.25 mg/g of adsorbent copper in the Langmuir model, and b (constant of adsorption tendency) was determined by 0.0127 L/mg.

**Table 3:** Langmuir–Freundlich adsorption isotherm constants for chromium (VI) adsorption.

Amount	Coefficient	Type
0.739	k (L/g)	Freundlich log (x/m) = log k + (1/n) log C_e_
1.19	n	
0.9561	R^2^	
45.25	X_m_(mg/g)	Langmuir 1/X = 1/X_m_ + (1/bX_m_) (1/C_e_)
0.0127	b(L/mg)	
0.9598	R^2^	
0.611	R_L_	

### Recreation and reuse of bee composite carcass and waste/polyaniline/polyethylene glycol

Reutilizing multiple adsorbents to remove water contaminants is ordinarily a great improvement because it can decrease the cost of synthesizing and purchasing the chemicals required to make or modify some adsorbents. Moreover, reusability restricts the disposal of adsorbents after a single-use^84^. As previous research explains, we can reuse some chromium adsorbents after a simple treatment with dilute solutions of sodium hydroxide, hydrochloric acid, or sulfuric acid^85–88^. According to the obtained results, for the initial solution of 50 ppm chromium, after the recovery of 10.58 ppm chromium was returned, which means about 41.85% desorption occurred. The recycled adsorbent was then re-used to remove chromium and increased the initial concentration of 50 mg/L chromium to 34.22 in similar conditions, and about 31.56% was removed.

## Conclusion

This study tested bee carcass composites and wastes and polyaniline with polyethylene glycol additive to remove hexavalent chromium from an aqueous medium. Ultimately, we recognized that the highest percentage of removal related to the polyethylene composite and bee carcass and waste in the presence of polyethylene glycol was 50.56% among the carcass and bee waste composites, which we then selected as the best adsorbent. The parameters influencing the adsorption process for polyethylene composite and bee carcass and waste in the presence of polyethylene glycol in the next step, which had the adsorption percentage increased for this composite by decreasing the pH, increasing the contact time, and increasing the adsorbent, and increasing the initial concentration of chromium. The highest percentage of adsorption was obtained when the pH was 2, the contact time was 120 min and the adsorbent value was 8 g/L and the initial concentration of chromium was 100 ppm. The most optimal removal percentage was achieved at the pH = 2, the contact time was 30 min, and the adsorbent value was 2 g /L, and the initial chromium concentration was 100 ppm. The outlining of the adsorption isotherms also revealed that the Langmuir equation had a better fit than Freundlich because of the higher R^2^. Ultimately, the results were achieved by reduction and desorption affirmed that the adsorbent could absorb continuously, and recreation and recovery performed.
